# Advances in Neurovascular Treatments

**DOI:** 10.1155/2014/641539

**Published:** 2014-06-05

**Authors:** Robert M. Starke, Stephen J. Monteith, Nohra Chalouhi, Dale Ding, Ricky Medel, David Hasan, Aaron S. Dumont

**Affiliations:** ^1^Department of Neurological Surgery, University of Virginia, P.O. Box 800212, Charlottesville, VA 22908, USA; ^2^Department of Neurosurgery, Swedish Neuroscience Institute, Seattle, WA 98122, USA; ^3^Department of Neurological Surgery, Thomas Jefferson University, Philadelphia, PA 19107, USA; ^4^Department of Neurological Surgery, Tulane University, New Orleans, LA 70012, USA; ^5^Department of Neurological Surgery, University of Iowa, Iowa City, IA 52242, USA

Neurovascular diseases compromise a heterogeneous group of disorders with a broad spectrum of phenotypes. Recent advances include greater understanding of both the genetic links and the basic mechanisms underlying the pathophysiology of cerebrovascular diseases. Improvements in neurovascular disease therapies have risen both from cell cultures and animal models of disease. Additionally, developments in technology have resulted in improvements in diagnosis, including a variety of advances in vascular imaging, medical therapies, and surgical and endovascular therapies. Recent randomized clinical trials have also helped to refine patient selection and optimal treatment modalities.

Stroke is a leading cause of morbidity and mortality. Three recent trials have challenged the use of endovascular therapy in acute cerebrovascular occlusion [1]. Limitations of these studies included lack of large vessel occlusions in many patients and the use of older thrombectomy devices. In the recent edition, S. Hann et al. demonstrate the benefits of next generation thrombectomy devices in clot removal. For patients with significant intracranial disease, recent trials have demonstrated that best medical therapy results in improved outcomes over intracranial stenting [2]. For patients who have failed best medical therapy, the optimal treatment options remain unclear. D. Ding et al. review potential options in the current edition, including balloon-mounted stents that may offer an effective alternative to self-expanding stents with lower rates of in-stent stenosis [3].

Cerebral arteriovenous malformations (AVMs) consist of an abnormal tangle of blood vessels that shunt blood directly from arteries to veins without an intervening capillary bed. The molecular mechanisms behind their development, progression, and hemorrhage remain incompletely defined, and further critical work is indicated for both risk stratification and the potential development of an alternative medical therapy. Recent studies have highlighted the altered role of Notch signaling pathway in AVMs [4], and the* in vivo* study by J. Tu and N. F. Jufri adds to the literature by demonstrating that wall shear stress likely contributes to AVM angiogenesis through activation of Notch. Further studies are necessary to determine how altered gene expression in critical vascular genes likely contributes to AVM instability and hemorrhage. Recent trials have challenged aggressive treatment of AVM patients at low risk for hemorrhage [5]. For patients with high risk features, intervention may be necessary. R. Dalyai et al. review clinical outcomes following radiosurgery with embolization in large AVMs.

Similarly, the molecular mechanisms underlying cerebral aneurysm formation and rupture remain unclear [6]. It has been known for some time that female gender is a significant risk factor for both aneurysm formation and subarachnoid hemorrhage. J. Tu et al. reviewed the role of estrogen receptors in signaling mechanisms in human cerebral vascular endothelial cells which may help to provide a preventative therapy for aneurysm progression. A number of trials have recently addressed the role of endovascular therapy in cerebral aneurysms [7]. For mycotic aneurysms, the optimal therapy is incompletely defined, and this area is eloquently reviewed by M. Zanaty et al. Following aneurysm rupture, vasospasm is a significant cause of morbidity and mortality. A number of preventative and treatment measures may be beneficial, including alterations in cerebral blood flow dynamics as assessed by D. K. Kung et al. and the efficacy of intra-arterial nimodipine as tested by S. Ott et al.

Neurovascular diseases are often associated with devastating outcomes. Progression from* in vitro*,* in vivo*, translational, and clinical studies has led to many new developments. Additionally, developments in technology have improved treatment outcomes. Although there have been numerous advances in treatment for neurovascular diseases, there remain many areas of uncertainty. Through this collection of papers, we hope to highlight areas of uncertainty, recent advances, and future clinical necessity.

*Robert M. Starke* *Stephen J. Monteith* *Nohra Chalouhi* *Dale Ding* *Ricky Medel* *David Hasan* *Aaron S. Dumont*
